# School eHealth education program Pakistan (eSHEPP): an exploratory qualitative study of stakeholder perspectives on design, barriers, and facilitators

**DOI:** 10.1186/s41043-025-01170-0

**Published:** 2025-11-26

**Authors:** Muhammad Shahid Khan, Aysha Almas, Zainab Samad, Kanecia Obie Zimmerman, Tazeen Saeed Ali

**Affiliations:** 1 Department of Medicine, Aga Khan University, Karachi, 74800 Pakistan; 2 Department of Pediatrics, Duke UniversityDuke University Medical Center, Durham, 27710 USA; 3 Duke UniversityDuke Clinical Research Institute, Duke University School of Medicine, Durham, 27701 USA; 4 School of Nursing and Midwifery, Aga Khan University, Karachi, 74800 Pakistan; 5 Department of Community Health Sciences, Aga Khan University, Karachi, 74800 Pakistan

**Keywords:** Adolescent health, Digital health, Health education, Schools, Pakistan, Noncommunicable diseases, EHealth, Qualitative research

## Abstract

**Background:**

Noncommunicable diseases (NCDs) are a growing health challenge in low- and middle-income countries (LMICs), including Pakistan. Adolescence is a critical period for shaping lifelong behaviors, yet school-based health education remains limited, and inconsistently implemented. Digital health interventions offer scalable opportunities, but their feasibility, sustainability, and cultural acceptability in LMIC school settings remain underexplored.

**Objective:**

This study examined barriers and facilitators to delivering the School eHealth Education Program Pakistan (eSHEPP) and explored stakeholder perceptions of its design, delivery, and content for adolescent NCD prevention.

**Methods:**

An exploratory qualitative design was applied in public secondary and higher secondary schools using purposive sampling. Data were collected through four Focus Group Discussions (FGDs) with students and teachers (*N* = 36) and 11 Key Informant Interviews (KIIs) with parents and administrators. KII and FGD guides were based on the Technology Acceptance Model and the Task–Technology Fit framework. Transcripts were thematically analyzed in NVivo v14 using a hybrid deductive–inductive approach. Credibility was supported through intercoder reliability (κ = 0.71) and stakeholder validation.

**Results:**

Major barriers included infrastructure gaps such as unreliable internet, electricity shortages, and lack of multimedia resources. However, students’ digital familiarity and widespread mobile access were strong facilitators. Parents, teachers, and administrators endorsed eSHEPP, noting students’ enthusiasm and the spillover of health knowledge to families. Stakeholders recommended a bilingual (Urdu/English), offline-accessible app with intuitive navigation, privacy safeguards, and interactive tools such as quizzes and rewards. Short Urdu videos with English subtitles, relatable scenarios, and student involvement were considered most engaging. Cultural sensitivities around mental health, gender norms, and substance use require careful framing. Sustainability was viewed as dependent on curriculum integration, teacher training, and institutional support.

**Conclusions:**

eSHEPP shows strong potential as a culturally sensitive, scalable, and pedagogically sound model for adolescent health promotion in LMIC schools. Addressing infrastructural gaps, ensuring policy integration, and promoting digital equity will be critical for long-term impact.

**Supplementary Information:**

The online version contains supplementary material available at 10.1186/s41043-025-01170-0.

## Introduction

Noncommunicable diseases (NCDs) and their risk factors are major global public health concerns, particularly in low- and middle-income countries (LMICs), where they account for a disproportionate burden of morbidity and mortality [1, 2]. Adolescents are especially vulnerable, as many risk behaviors that contribute to NCDs, including physical inactivity, substance use, poor diet, and overweight or obesity, are established during this stage of life [3–5]. These behaviors have long-term consequences, with research showing that a large share of premature adult deaths are linked to habits formed in adolescence [4, 5]. Recognizing this, the World Health Organization (WHO) has emphasized NCD prevention among adolescents as a public health priority and called for innovative, scalable approaches to reach young people effectively [6, 7].

Schools are uniquely positioned to promote adolescent health. As a primary setting where young people spend much of their time, schools offer a platform for structured health education, skill-building, and behavior change [8, 9]. Evidence from LMICs shows that school-based interventions can successfully improve students’ nutrition, physical activity, and other health-related behaviors [10–12]. However, in Pakistan, school health education remains limited and fragmented. Health topics are infrequently integrated into the formal curriculum, and when present, they often rely on didactic lectures that emphasize information transfer rather than student engagement or skill development [13, 14].

Several systemic barriers further limit the effectiveness of health education in Pakistani public schools. These include inadequate infrastructure, limited teaching resources, and a shortage of instructors trained in adolescent health [15]. Cultural sensitivities also constrain discussions on topics such as mental and reproductive health and gender equity, leaving many students without access to accurate information or supportive dialogue [14, 16]. Consequently, adolescents often lack the knowledge, motivation, and confidence needed to make informed health choices, and schools remain an underutilized platform for NCD prevention.

Digital interventions offer an innovative, accessible, and cost-effective way to address these gaps. With the rapid growth of mobile phone and internet use in Pakistan [17], digital platforms provide promising opportunities to deliver engaging, age-appropriate, and culturally relevant health content [18, 19]. Children and adolescents are already active users of mobile applications, social media, and online platforms, which have been leveraged globally for peer-to-peer education, health promotion, and mental health support [20–23]. Emerging evidence shows that eHealth interventions can enhance health literacy and encourage positive health behaviors, especially in resource-constrained settings [18, 21, 22, 24–28]. However, the success of such programs depends on how well they reflect users’ daily realities, technological access, and sociocultural contexts. Interventions developed without this grounding risk a “design–reality gap,” leading to low engagement and sustainability [29–31], while those shaped by stakeholder input achieve stronger engagement and more sustainable impact [29, 32, 33].

Formative qualitative research is therefore essential to guide the design and delivery of school-based eHealth programs in Pakistan. By capturing the perspectives of key stakeholders, including students (end users), teachers (program implementers), parents (caregivers), and school administrators and policymakers (decision-makers), researchers can ensure that interventions are contextually appropriate and practically feasible within the constraints of the public education system.

To respond to this need, the research team initiated the development of the School eHealth Education Program Pakistan (eSHEPP), a digital, school-based intervention designed with stakeholder input to improve adolescents’ health literacy and reduce modifiable NCD risk behaviors. The detailed objectives and methodological framework for eSHEPP are described in a previously published protocol [34]. This exploratory qualitative study examines barriers and facilitators to implementing eSHEPP in public secondary and higher secondary schools and explores stakeholder perceptions of its design and content. To our knowledge, this is the first study in Pakistan to qualitatively investigate stakeholder perspectives on a school-based eHealth program for adolescents. Findings will inform refinements to eSHEPP and support the integration of digital health education into national education and public health systems, offering lessons for other LMICs.

## Methods

An exploratory qualitative study was conducted across public secondary and higher secondary schools in Karachi, Pakistan, to identify barriers and facilitators affecting the delivery of eSHEPP in secondary and higher secondary schools, and to capture stakeholders’ perspectives on the program’s design and content. The study was conducted in Karachi rather than nationwide, aiming to explore why and how contextual, infrastructural, and cultural factors influence these barriers, facilitators, and stakeholder perceptions. Public schools were chosen because they often face resource constraints, such as limited access to digital infrastructure, teaching aids, and health education materials, making them a realistic context for developing school-based eHealth interventions. The research team sought to tailor eSHEPP to the needs and expectations of these schools so that, once optimized, it could be scaled more effectively across urban public schools in Pakistan. Karachi was selected as the study site because it is the country’s largest metropolitan area, representing diverse socio-economic, cultural, and educational contexts within both urban and peri-urban settings [35].

### Conceptual framework for developing key informant interviews (KIIs) and focused group discussion (FGD) guides

**Fig. 1:**
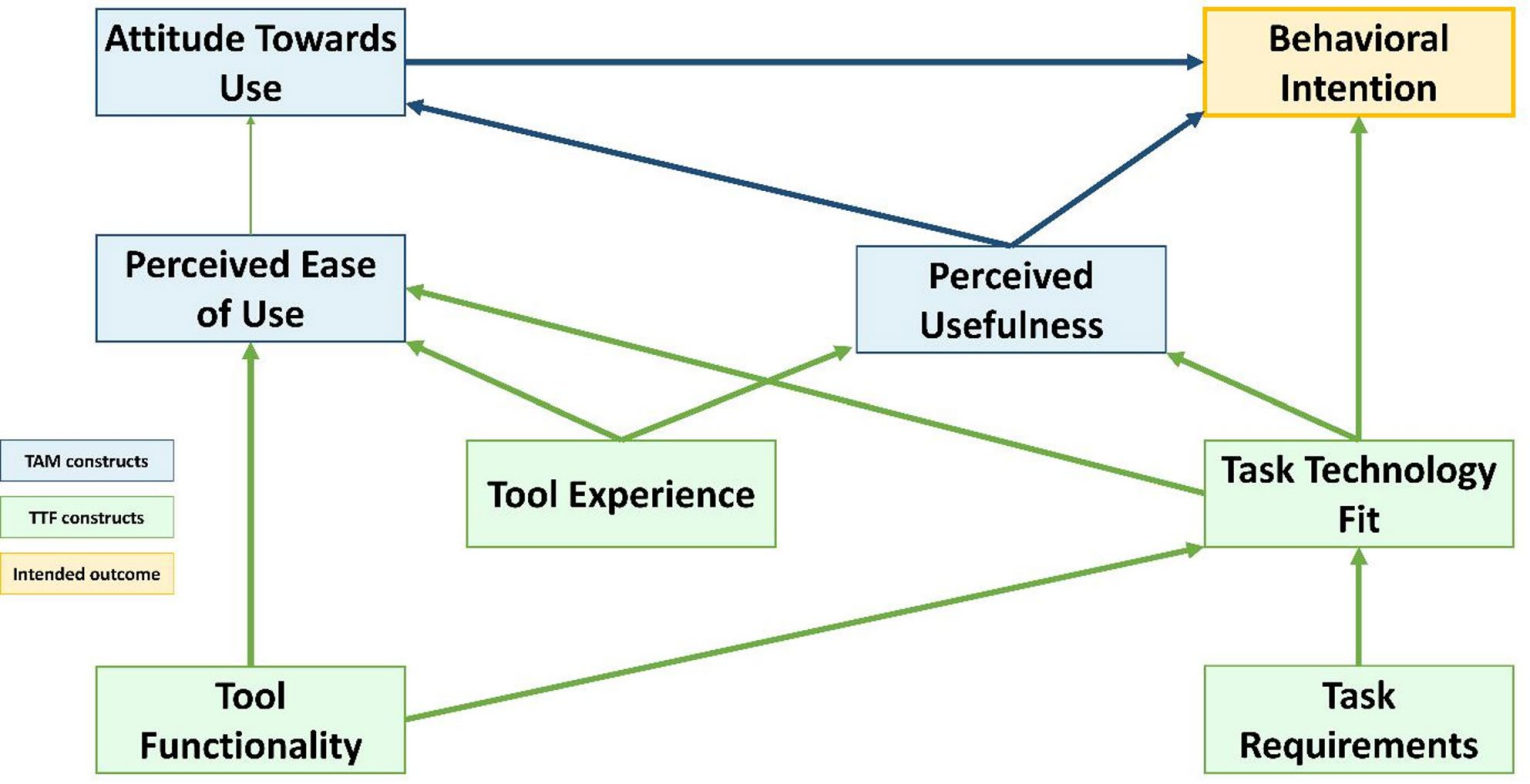
Integrated model of TAM and TTF

This exploratory qualitative study was guided by an adapted conceptual framework that integrates the Technology Acceptance Model (TAM) and the Task–Technology Fit (TTF) [36]. The framework was used to explore users’ perceptions of the eSHEPP program’s design, with a particular focus on perceived usefulness, perceived ease of use, and attitudes toward use. These components provided a structured approach to understand key user insights relevant to the effective development of the eSHEPP intervention. Figure 1 shows the conceptual framework guiding the development of the FGD and KII guides, which are provided in S5 File.

The integration of TAM and TTF was intentionally chosen to provide a comprehensive lens for understanding both the individual and contextual factors influencing stakeholders’ perceptions of eSHEPP. TAM helps explain user acceptance by examining perceived usefulness and ease of use, while TTF focuses on the alignment between technological features and the tasks users aim to perform. Together, these frameworks complement each other, TAM captures behavioral and attitudinal factors driving adoption, whereas TTF contextualizes these within the educational setting and practical delivery environment. This combination allowed for a nuanced interpretation of how eSHEPP’s design and delivery could be optimized to fit user needs and school contexts.

### Sampling technique and participants

A purposive sampling strategy was employed to ensure diverse representation across stakeholder roles, experience levels, and availability. Participants were recruited through school principals, who facilitated initial contact with students, teachers, and parents, while school administrators were directly approached for interviews.

Inclusion criteria for participants were as follows: students in grades 9–12, teachers with at least six months of teaching experience in relevant schools, parents or guardians of enrolled students, and school administrators, including principals, vice principals, District Education Officers (DEOs), and officials from the Provincial Department of Education.

Four FGDs were conducted: two with students (one male, one female) with 10 participants in each group, and two with teachers (one male, one female) with 8 participants in each group. In addition, 4 KIIs were conducted with parents and 7 KIIs with school administrators to gather in-depth insights from those in caregiving and leadership roles. In total, 47 participants were included: 20 students, 16 teachers, 4 parents, and 7 school administrators. Participants for FGDs and KIIs were purposively selected to ensure diversity in gender, experience, and school type. School principals facilitated the initial identification of potential participants, and the research team confirmed eligibility based on the inclusion criteria. One parent and two administrators declined participation due to time constraints. No individuals other than participants and researchers were present during data collection. There was a slight deviation from the participant numbers proposed in the study protocol, as additional interviews were conducted with school administrators to ensure data saturation. These adjustments were guided by methodological rigor rather than participant availability and did not affect data quality or thematic comprehensiveness. Detailed demographic characteristics of the study sample are provided in S3 File (Tables S4–S7).

## Data collection

Semi-structured interview guides were developed using the integrated TAM–TTF framework and organized into four sections: general perceptions of digital health applications (TAM: perceived usefulness and ease of use), barriers to implementation (TTF: students’ learning.

needs, engagement, and contextual challenges), facilitators to implementation (factors supporting delivery), and program content and design (perspectives on videos and application features). The guides were initially prepared in English, translated into Urdu, and pilot tested (two FGD and two KIIs) to ensure clarity and cultural relevance. Minor adjustments were made to address general confusion around a few Urdu terms; no other substantive changes were required, as the guides were otherwise well understood and effective for use in the main study. At the time of data collection, participants had no direct exposure to eSHEPP; their perspectives were gathered to guide program design and adaptation before school-based delivery.

FGDs, lasting 45–60 min, and KIIs, lasting 20–30 min, were conducted sequentially across participating public secondary and higher secondary schools in Karachi, Pakistan. In coordination with the Directorate of Education, Karachi, two secondary and two higher secondary schools were identified for data collection. Separate FGDs were held in boys’ schools with male students and teachers, and in girls’ schools with female students and teachers to ensure cultural appropriateness and participant comfort. Parents of participating students were invited to their respective schools for KIIs, while KIIs with school administrators from various institutions across Karachi were conducted in their offices. Data collection took place within school premises, primarily in activity rooms, classrooms, or stakeholder offices, to ensure privacy, comfort, and minimal disruption to school routines. Each session began with informal conversation to build rapport and explain the study purpose, and discussions were audio-recorded and supplemented with field notes. Written informed consent was obtained from all participants, with both parental consent and student assent secured for those under 18.

### Research team and reflexivity

All FGDs and KIIs were conducted by the lead researcher (MSK, male, PhD Candidate (Population and Public Health, Aga Khan University), formally trained in qualitative methods with prior community-based research experience. No prior relationships existed with participants; contact was facilitated through school administrators. For cultural sensitivity, the male researcher conducted FGDs and KIIs with male participants, while a trained female research assistant facilitated those with female participants. Reflexivity was maintained through ongoing debriefs with the research team to critically examine assumptions and potential biases.

### Data management and analysis

Data collection continued until thematic saturation was reached. Saturation was operationally defined as the point at which no new codes, concepts, or subthemes emerged from two consecutive FGDs or KIIs. The research team met regularly during data collection to review preliminary coding summaries and assess emerging themes. Thematic saturation was confirmed after conducting all planned FGDs and KIIs (with a total of 47 participants), as no new insights or variations were identified in the final discussions and interviews. Audio recordings were transcribed verbatim and cross-verified by the lead researcher within two weeks. Data were analyzed using a hybrid coding approach: deductive coding based on TAM–TTF constructs and inductive coding for emergent themes.

The codebook underwent six iterative development cycles over three months, with team discussions refining parent–child codes and ensuring alignment with the integrated TAM–TTF framework (see S1 File: Tables S1–S3). Two independent coders analyzed the transcripts. Intercoder reliability was assessed on a subset of transcripts (25%), yielding substantial agreement (Cohen’s κ = 0.71, *p* <.001). A detailed coding tree is available in S3 File (Figure S2). Thematic validation was achieved through team discussions, and representative quotes were selected to illustrate key findings. To enhance credibility, a stakeholder validation meeting was conducted where preliminary findings were presented and discussed, allowing participants to provide feedback on interpretation. Data were managed and analyzed using NVivo v14. The study adhered to the Consolidated Criteria for Reporting Qualitative Research (COREQ) checklist throughout (see S4 File).

### Ethical approval

The study received ethical approval from The Aga Khan University Ethical Review Committee (Ref No. 2023-9277-27367). Any significant amendments were subject to approval by the project’s steering committee and re-submission to the ethics committee.

## Results

**Fig. 2:**
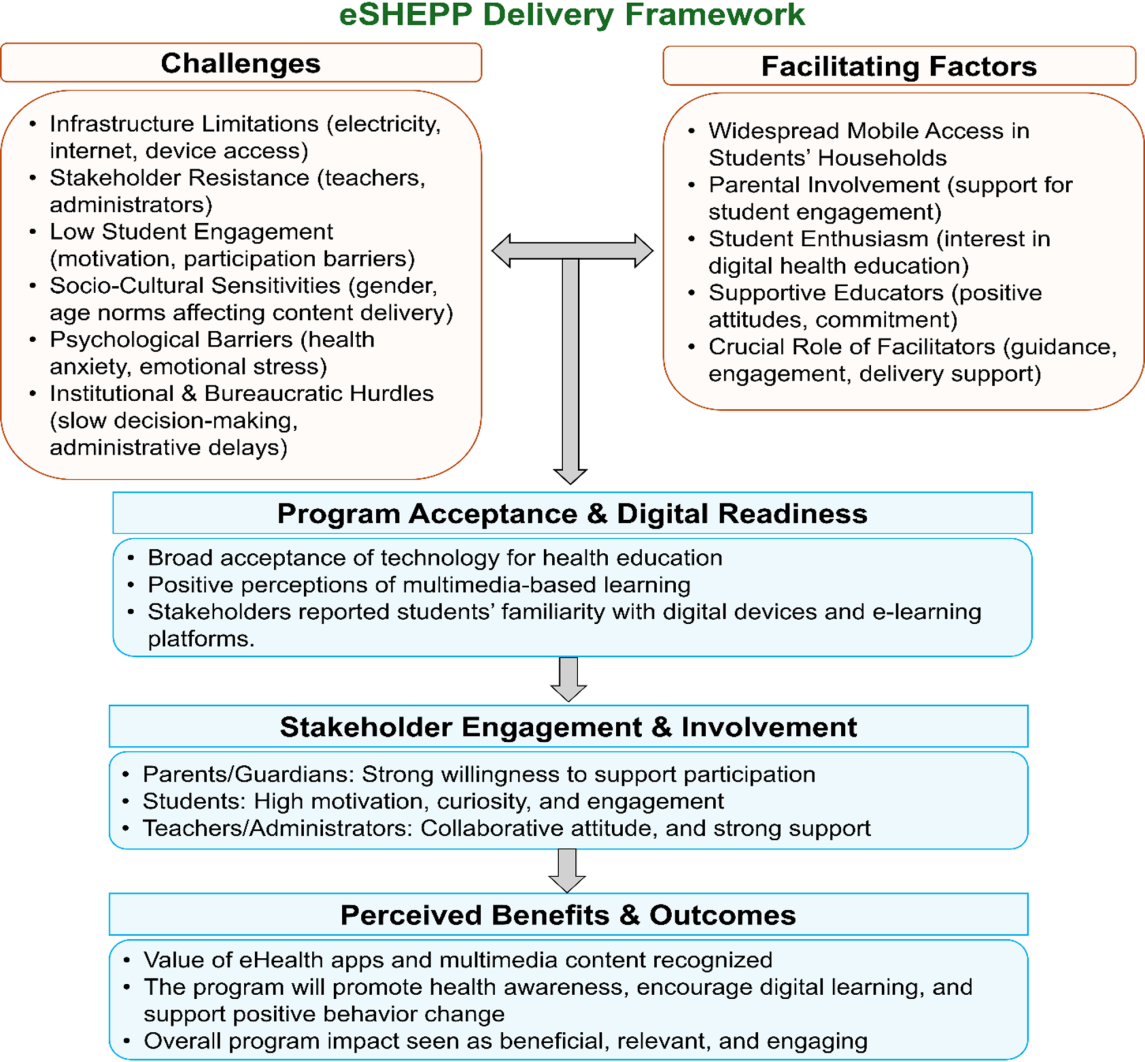
eSHEPP delivery framework for school students aged 13–18 years

Stakeholders identified several key challenges to eSHEPP implementation, including limited infrastructure, resistance to change, sustaining student engagement, and socio-cultural sensitivities. Enabling factors included widespread mobile phone access, parental encouragement, student motivation, and strong teacher and administrator support. Participants emphasized that active involvement of all stakeholder groups was essential for success and demonstrated readiness for digital learning through confidence with multimedia tools and technology. These findings collectively informed the proposed eSHEPP delivery framework (Fig. 2).

### Anticipated challenges

Stakeholders highlighted multiple barriers to program delivery. Infrastructural limitations were most frequently cited, including lack of multimedia equipment, unreliable electricity, and poor internet access. As Administrator 2 explained, *“Most government schools do not have multimedia systems*,*”* while Administrator 1 added, *“A dedicated activity or multimedia room is essential… not all schools have these resources.”* Electricity and connectivity were recurring concerns: *“There is a significant issue with electricity”* (Administrator 7), and teachers estimated that *“Only five to ten% of schools might have [internet access]”* (FGD-T1).

Resistance among senior staff also emerged as a challenge. Administrator 1 remarked, *“Some older teachers are rigid and disinterested*,* viewing sessions as a waste of time.”* Similarly, principals’ support varied: *“Some principals cooperate*,* others do not. Convincing them is part of your role”* (Administrator 2).

Maintaining consistent student attendance and motivation was another barrier. Administrator 4 highlighted absenteeism: *“Absenteeism is a significant challenge… extraordinary steps are needed to ensure attendance.”* Administrator 5 added, *“Children are generally not inclined to participate in such activities nowadays; you will need to motivate them effectively.”*

Cultural sensitivities and social boundaries, particularly related to gender norms and religion, were common concerns. Parent 3 explained, *“In our Pakistani culture*,* there are certain boundaries… doing so can create awkwardness*,* especially for females.”* Administrator 3 noted, *“Some matriculation students may prefer not to receive information in a particular way due to cultural practices*,* such as wearing a niqab.”*

Participants also warned that poorly framed health messages might cause anxiety or fear. Administrator 3 stated, *“Raising awareness may generate anxiety*,* and you need to know how to deal with it.”* Teachers similarly noted, *“Medical information can cause unnecessary worry… it is important that students act appropriately without becoming anxious”* (FGD-T2).

Administrative and external disruptions were also mentioned. Parent 3 highlighted bureaucratic hurdles: *“Approval from higher authorities is required*,* but once secured*,* they can influence students.”* Teachers also warned of interruptions from unplanned events: *“A sudden government program could halt your entire project”* (FGD-T2).

### Facilitating factors

Despite challenges, stakeholders identified multiple enablers for eSHEPP success. Access to digital devices and connectivity was widely reported, even in low-income families. Administrator 6 remarked, *“Smartphones have reached even the poor… I have seen laborers using them.”* Where broadband was lacking, families relied on affordable mobile data: *“People use mobile data*,* as affordable packages are available”* (Administrator 6). Teachers often bridged digital gaps by contributing their own equipment: *“We brought our own laptops in previous initiatives… teacher interest is crucial”* (Administrator 7).

Parental engagement and awareness emerged as strong facilitators. Administrator 1 described awareness as “medium,” noting that parents frequently raised concerns about unhealthy food and its links to addiction. Parent 2 emphasized active involvement: *“We even discuss [anti-tobacco ads] with our children*,*”* and further argued, *“It should have been done at the government level*,* it will be very beneficial.”*

Student interest and participation were also strong enablers. Administrator 1 observed, *“Students… use technology more.”* Students themselves stressed the value of multimedia content: *“These videos will help us learn things not covered in our curriculum”* (FGD-S2). Teachers noted the importance of peer learning: *“If some children don’t understand*,* others help them… they often became facilitators themselves”* (FGD-T1).

Support from teachers and administrators was seen as essential, with the program described as complementary to existing curricula. Teachers explained, *“This complements our science and life skills classes and saves lesson prep time”* (FGD-T1). Administrators recalled prior positive experiences: *“We used to show health content with projectors… it had a strong impact”* (Administrator 2). Teachers also highlighted feasibility: *“Taking 20 to 30 minutes weekly is manageable”* (FGD-T2).

The facilitator’s role was emphasized, particularly in resource-limited schools. Administrator 2 noted, *“Most government schools lack multimedia*,* but your representative could bring it and prepare them.”* Students were described as especially receptive to external facilitators: *“Students pay more attention when someone from outside visits… change matters”* (Administrator 3). A hybrid teacher–facilitator model was preferred: *“Teachers should stay involved*,* so students respond more positively”* (Administrator 5).

### Stakeholder engagement and involvement

Parental involvement varies by socio-economic context. Urban families were generally more engaged, while rural and low-income households faced barriers due to livelihood pressures. As Administrator 6 explained, *“Parents are too focused on earning a livelihood to pay attention to such matters.”* Shared device use, however, encouraged indirect participation: *“When students watch videos*,* they do so with parents… who automatically get involved”* (FGD-T1).

Student expectations centered on short, interactive, and emotionally relatable content. One student remarked, *“If there’s a sad scene*,* there should be sad music*,* like in movies”* (FGD-S1). Students also valued creating content, with Administrator 3 noting, *“They get extremely excited seeing their own videos… parents also express satisfaction.”*

Teacher and administrator engagement was seen as critical for sustaining eSHEPP. Parent 4 described the cascade of influence: *“If the principal understands the message*,* they guide teachers*,* who guide students*,* and students influence parents… a motivated principal can motivate both teachers and parents.”* Teachers emphasized their role in continuity: *“Teachers can register new students and explain the program each year”* (FGD-T1). School Management Committees (SMCs) were also viewed as effective platforms. Administrator 1 explained, *“If the HM*,* teachers*,* and parents are involved*,* 50% of success is already achieved.”* A triangular model linking teachers, students, and parents was emphasized: *“When teachers are informed*,* students learn; when students learn*,* it reaches the parents”* (FGD-T1).

Stakeholders highlighted the importance of teacher training for sustainability. Administrator 1 suggested, *“It’s better to train teachers as master trainers and empower them to continue… If school health is part of teacher training*,* students will more readily accept it.”* Teachers echoed this: *“With proper training and a user-friendly guide*,* we can run the program independently after it ends”* (FGD-T2).

### Program acceptance and readiness for digital health education

Stakeholders expressed strong support for digital tools in health education, citing their accessibility, flexibility, and alignment with students’ media habits. Administrator 3 emphasized, *“Some things can’t be conveyed verbally; multimedia plays a key role.”* Parents echoed this enthusiasm, highlighting feasibility and inclusivity: *“Almost every second household has a phone… an app is definitely needed”* (Parent 2). Parent 3 added, *“Showing videos in schools is a great idea; real-life examples are more effective than textbooks.”*

Multimedia-based content, particularly short videos, dramatizations, and animations, was viewed as engaging and more effective than textbook learning. Administrator 7 noted, *“Children are fed up with textbook knowledge. They want to see everything practically.”* Parent 2 explained, *“When you show them a video… it becomes much clearer. If you just give them the facts to memorize*,* it is difficult for them.”*

Students across urban and rural schools were described as digitally literate and confident in using smartphones, apps, and online platforms. Teachers confirmed, *“They use gadgets a lot; using apps won’t be difficult.”* (FGD-T1), while also noting prior use for formal learning: *“Their classes were on Google Classroom”* (FGD-T2). Another teacher observed, *“You can assume 100% know how to use digital apps”* (FGD-T2).

Although students and teachers frequently engaged with digital platforms for entertainment or informal learning, structured health education apps were largely unfamiliar. As students shared, *“I used a health and care app… it showed how to maintain one’s body and environment”* (FGD-S1). Others remarked on the lack of local tools: *“I don’t think such an app exists in Pakistan… maybe in America or England”* (FGD-S2).

### Perceived benefits and motivation

Participants praised the eHealth app as a flexible, private learning tool with advantages over one-time sessions. Students valued its repeatability: *“The app would be better; we can access information anytime”* (FGD-S2). Parents emphasized institutional credibility: *“We can’t verify other platforms*,* but if this is from an institution*,* it’s excellent”* (Parent 4). The app also supported home-based learning, with Administrator 7 noting, *“The child will educate parents at home… many concerns will be addressed there.”*

Health videos were seen as powerful drivers of behavior change, with visual methods preferred over verbal instruction. As teachers noted, *“If they only hear*,* they may forget; if they see*,* they remember; if they do it*,* it becomes expertise”* (FGD-T2). Videos were also considered easy to integrate into classes, as Administrator 1 suggested: *“If we align them with the science period*,* it will positively influence behavior and attitudes.”*

The program was viewed as timely and culturally acceptable, addressing urgent health needs while reinforcing academics. Parent 4 affirmed, *“There will be no cultural or social barriers… students*,* teachers*,* and principals will unite to make it successful.”* A strong ripple effect on families was anticipated, with students seen as effective conduits to households. Administrator 6 shared, *“If children are told*,* ‘Tell your parents…’ education can reach families.”* Participants also stressed the need for scaling and institutionalization. Administrator 2 concluded, *“Your program should succeed; the government should adopt it through the education department.”*

### Stakeholder recommendations for ehealth application and health-promoting videos

**Fig. 3:**
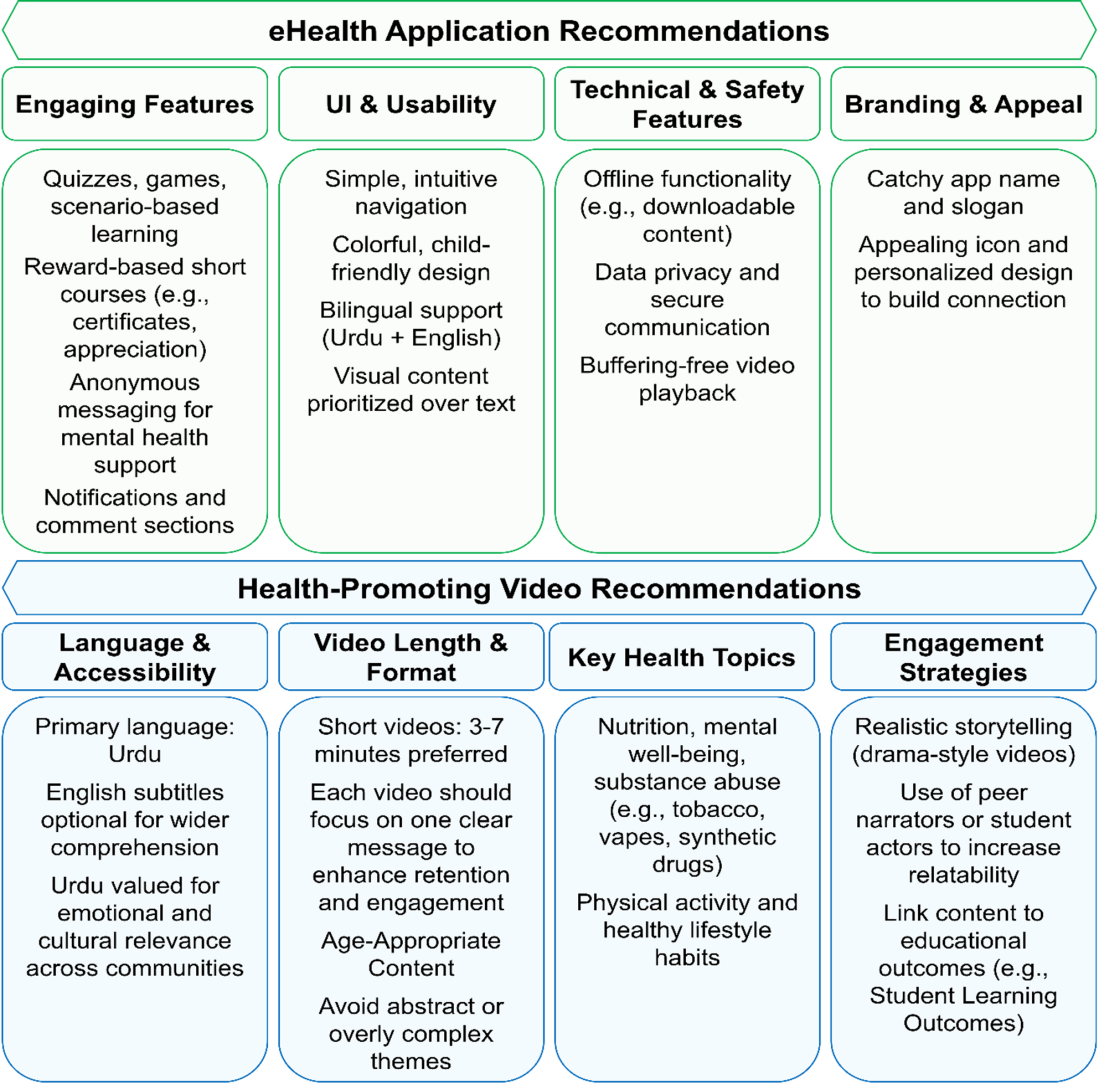
Stakeholder Recommendations for eHealth App and Health-Promoting Videos

Stakeholders recommended a simple, engaging app (English with optional Urdu) with intuitive navigation, interactive features, offline access, privacy safeguards, and appealing branding. They emphasized short Urdu videos with English subtitles on key health topics, delivered through relatable storytelling and student actors to enhance engagement. These recommendations are summarized in Fig. 3.

### Recommendations for the eHealth application

Stakeholders emphasized the value of interactive and reward-based features to sustain engagement. Students proposed, *“There should be multiple-choice questions based on small examples”* (FGD-S1), while Administrator 4 suggested, *“Offer certificates or appreciation letters for participation.”* Gamification was highlighted as a strong motivator: *“Completing one level unlocks the next; we can collect stars”* (FGD-S2). To support mental health, anonymous messaging was proposed; as Administrator 1 noted, *“If problems exist at home*,* students may not talk to family… it’s easier to message someone anonymously.”* Parents also recommended notifications and comment sections to enhance interaction: *“The comment section is very important*,*” and added*,* “A notification option would be great”* (Parent 1). Branding was seen as critical, with Administrator 3 advising, *“The app’s name should feel personal… the slogan should be catchy*,* and the icon appealing.”*

Ease of use was identified as essential, with calls for a simple, child-friendly interface, intuitive navigation, and bilingual support. Administrator 4 emphasized, *“The application should be user-friendly… Keep the language simple.”* Visual elements were preferred over text, as teachers noted, *“Use pictures; they capture children’s attention better”* (FGD-T1).

Participants identified technical and safety concerns. Students suggested a download option to address connectivity issues: *“Students suggested a download option to address video buffering”* (FGD-S2). Concerns were also raised regarding child safety in communication features. Administrator 1 asked, *“How is safety ensured when a child communicates with someone from outside?”*

### Health-promoting videos recommendations

Urdu was consistently identified as the preferred medium, valued for accessibility and emotional resonance. As Administrator 6 emphasized, *“Definitely in Urdu… no need for regional languages.”* English subtitles were recommended for higher-tier or English-medium schools.

Stakeholders agreed that videos should be short (3–7 min) to sustain attention. Students suggested, *“Videos should be around 5–6 minutes… explain one point clearly”* (FGD-S1).

Core content priorities included nutrition, mental health, substance abuse, physical activity, and healthy habits. Teachers highlighted dietary concerns: *“Children now consume sugary drinks and colas… harmful to health”* (FGD-T2). Administrator 1 stressed, *“Mental health is essential*,* especially at puberty.”* Substance abuse emerged as a major concern. Parent 3 urged, *“Add drugs alongside tobacco… include alcohol and other addictive substances.”*

In terms of format and style, participants recommended age-appropriate dramatizations. Administrator 1 noted, *“Cartoons suit younger students… for grades 9–12*,* use real characters or a mix.”* Administrator 2 added, *“It should be a drama or story-type video explaining diseases and prevention.”*

Stakeholders stressed the importance of clear goals, interactivity, and educational alignment to sustain engagement. Administrator 3 explained, *“You need a well-planned strategy… if it relates to their school or locality*,* they become more invested.”* Administrator 1 cautioned, *“If they see it as mere entertainment*,* they will treat it like a cartoon and forget it. Link it to their learning and SLOs [Student Learning Outcomes]*,* and they will take it seriously.”* Teachers also emphasized age-appropriateness: *“If it’s too complex*,* they won’t grasp it and will lose interest”* (FGD-T1).

**Fig. 4:**
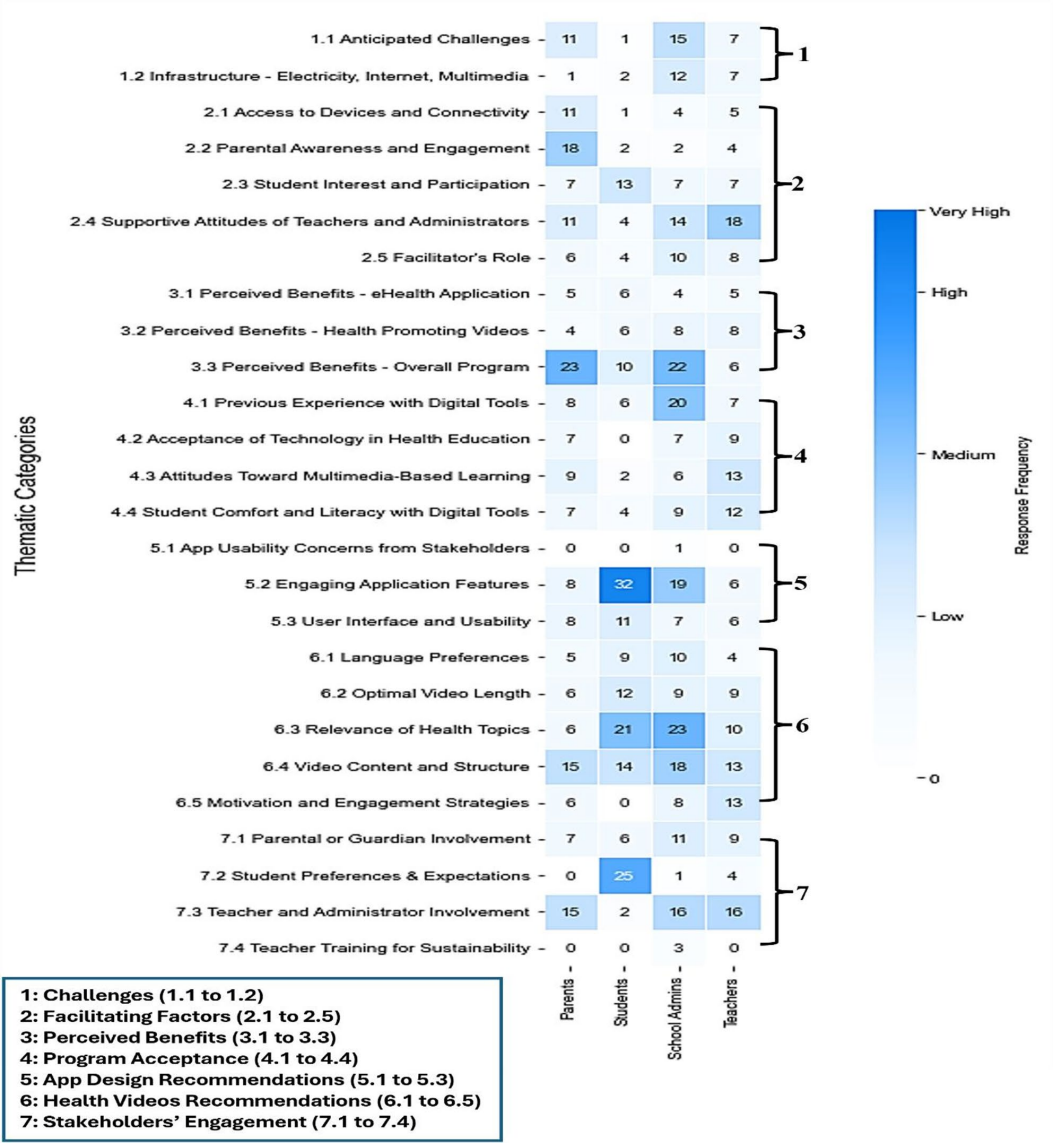
Stakeholder responses to eSHEPP themes

Figure. 4 presents response frequencies from parents, students, teachers, and administrators across seven themes (challenges, facilitators, perceived benefits, program acceptance, stakeholder engagement, and recommendations for app design and health videos). Teachers emphasized support and involvement; students prioritized app design and video content; administrators and parents focused on program benefits. While qualitative analysis primarily emphasizes thematic depth, frequencies are presented here to illustrate the relative salience of themes across stakeholder groups, enhancing interpretive clarity without implying statistical inference [37]

## Discussion

Stakeholders noted key challenges to the delivery of eSHEPP, including inadequate infrastructure, resistance to change, and socio-cultural sensitivities, but also identified strong enabling factors such as widespread mobile access, parental encouragement, motivated students, and supportive teachers and administrators. They consistently emphasized the importance of stakeholder engagement and highlighted students’ readiness and enthusiasm for digital learning, expressing confidence in the potential of multimedia and technology-based tools for health education.

Stakeholders recommended a user-friendly and visually engaging eHealth app with Urdu and English language options, simple navigation, offline functionality, privacy protections, and interactive features such as quizzes and rewards, alongside appealing branding. For videos, they preferred short Urdu-language segments with English subtitles, covering topics such as nutrition, mental health, physical activity, and substance use. They suggested using relatable storytelling and student participation to improve engagement and comprehension.

### Infrastructure, access, and digital engagement

Stakeholders identified infrastructural barriers such as unreliable electricity, limited internet connectivity, and insufficient multimedia equipment. Similar challenges are reported in other low-resource settings, including Pakistan, where few public schools have adequate digital equipment or connectivity [38–40]. This aligns with national assessments of digital health readiness, which highlight systemic challenges including insufficient infrastructure, limited technical capacity, and fragmented implementation [41]. Our findings indicate that these gaps persist at the school level, constraining both feasibility and scalability of digital health interventions.

Despite these barriers, participants reported a high level of digital literacy and familiarity among both students and teachers. Mobile phone access, especially smartphones, was common even in low-income households. Students frequently accessed digital content, and teachers often used personal devices to overcome institutional gaps. These findings mirror experiences from LMICs such as Zimbabwe and India, where mobile-first strategies and informal device-sharing networks compensate for weak institutional infrastructure [42, 43]. This contrast between limited institutional resources and strong individual digital competence underscores the potential for mobile-first eHealth programs in Pakistan, while emphasizing the need for systemic ICT investments.

### Stakeholder involvement and community acceptance

The commitment to active stakeholder participation, including students, teachers, parents, and administrators, was a defining strength of eSHEPP and a major contributor to its positive reception. Students described the digital sessions as enjoyable and non-intrusive, teachers acknowledged their educational value, and parents endorsed the initiative, demonstrating broad-based support within the school community. This multi-level buy-in echoes Langford et al. (2014), who found that engagement from students, staff, and families is vital for the sustainability of school-based health interventions [44]. Comparable evidence from South Asia, such as the POD Adventures digital mental health pilot in India, shows that co-design with teachers and students enhances feasibility, acceptability, and engagement [42, 45]. Our study is the first in Pakistan to report such comprehensive stakeholder endorsement of a digital school-based health initiative, establishing a solid foundation for institutionalization and scale-up.

Teachers and administrators played a pivotal role in supporting implementation and motivating student participation. This aligns with Rogers’ Diffusion of Innovations theory, which identifies early adopters as key influencers in promoting new educational technologies [46]. Building on this momentum, integrating digital health into pre-service and in-service teacher training could strengthen sustainability and ensure program continuity.

Parental support was particularly strong in urban settings. Parents expressed enthusiasm for app-based learning and recognized its potential to improve family health literacy. Because mobile devices are often shared within households, students frequently acted as conduits of information, transferring health knowledge to family members. This reflects relational models of health education in LMICs, where adolescents function as “knowledge brokers” extending program reach into families and communities [47]. These findings suggest that school-based digital interventions may also operate as family-centered health promotion tools, amplifying their broader social impact.

### Cultural appropriateness and sensitivity

Socio-cultural sensitivities, particularly around mental health, substance use, and gender norms, emerged as recurring themes. Evidence consistently highlights the need for culturally sensitive framing and language for adolescent health interventions in conservative settings. The WHO’s 2020 youth-centered digital health framework similarly advocates adapting content to local cultural contexts [48]. In Pakistan, school-based psychological programs that reflect family and gender dynamics have shown greater feasibility than non-adapted versions [49]. Likewise, South Asian studies indicate that linguistically and culturally tailored digital materials enhance accessibility, trust, and student participation [50]. Comparable barriers in Bangladesh and India, such as limited opportunities for girls’ physical activity due to restrictive norms, underscore the importance of gender-sensitive and community-supported approaches [45, 51, 52].

Stakeholders preferred short, Urdu-language videos featuring realistic scenarios, dramatizations, and peer involvement, which were perceived as more relatable and emotionally engaging than abstract or technical presentations. They also emphasized avoiding fear-based messaging, preferring constructive and solution-oriented narratives. These preferences align with global evidence that culturally adapted health communication strategies improve both comprehension and engagement [53, 54]. By foregrounding cultural sensitivity in both language and pedagogy, this study illustrates how locally grounded eHealth approaches can enhance acceptance and impact among adolescents.

### Pedagogical value and student empowerment

Multimedia tools, such as videos, games, and quizzes, were widely recognized for their pedagogical effectiveness. Students engaged more deeply with interactive and story-driven content, and teachers observed greater motivation and knowledge retention compared to traditional textbook-based instruction. Evidence supports these findings: Donkor (2011) reported high satisfaction with video-based materials [55], and more recent studies highlight that interactive formats increase learning engagement and satisfaction [56, 57]. These findings align with learner-centered theories that emphasize relevance, interactivity, and active participation [58, 59].

Importantly, students emerged as active contributors, often sharing health information with peers and family members. This ripple effect extended the program’s reach, a pattern observed in LMIC interventions where adolescent co-creation fosters ownership and sustainability [42, 60–62]. Thus, students acted not only as learners but also as peer educators, reinforcing their role in amplifying program impact beyond the classroom.

#### Sustainability, scale, and system integration

While stakeholders valued eSHEPP’s contributions to adolescent health education, its long-term success depends on institutional integration. Sustained support from school leadership and teaching staff will be essential for continued implementation. Stakeholders emphasized the importance of endorsement by education authorities and alignment with national curricula. In comparable initiatives such as Pakistan’s Hayat digital health system, policy integration and government funding were crucial for scaling beyond pilot stages [63].

Although high mobile phone penetration provides a viable delivery channel, digital inequities must be addressed to ensure equitable access. Recommended strategies included offline functionality, downloadable modules, and hybrid facilitation models pairing teachers with trained assistants, particularly in under-resourced areas. Recent analyses of digital health in Pakistan also stress the need to overcome infrastructure deficits, unreliable connectivity, and weak governance to enable scalability [41].

Training select teachers as program champions, supported by simple digital aids, was viewed as key for sustainability. Consistent with LMIC experiences, long-term success requires government endorsement, integration into education policy, and multi-stakeholder partnerships [64, 65]. Our findings highlight three core prerequisites for sustainable scale-up: investment in ICT infrastructure, integration into teacher training, and alignment with national education strategies.

### Limitations and future directions

While qualitative design provided in-depth contextual insights, it also introduced certain limitations. The sample, though diverse, may not fully represent the full geographic and socio-economic variation across Pakistan. Additionally, while some stakeholders perceived early indications of improved awareness and motivation among students, this qualitative phase did not measure behavioral outcomes; therefore, such interpretations should be viewed as perceptions rather than verified changes. This study represents the formative qualitative phase of a broader mixed-methods project designed to inform the development and delivery of eSHEPP. It specifically explored barriers, facilitators, and stakeholder perspectives to guide program refinement and implementation in public secondary and higher secondary schools.

Future research should examine program applicability in remote and marginalized areas and employ quantitative and longitudinal designs, such as randomized controlled trials, to assess effectiveness and sustained behavioral outcomes. Integrating usage analytics and real-time feedback mechanisms may further enhance responsiveness and iterative improvement. Overall, these findings offer transferable lessons for other LMICs, underscoring the importance of stakeholder-driven, contextually grounded digital health interventions to bridge the “design–reality gap” and strengthen adolescent health promotion.

## Conclusion

Findings from this exploratory qualitative study suggest that the eSHEPP holds promise as a culturally appropriate, feasible, and pedagogically engaging approach to school-based health education in Pakistan. Stakeholders, including students, teachers, parents, and administrators, expressed strong support for the program’s content and delivery, particularly its use of multimedia and interactive tools that enhance engagement and align with modern learning approaches. Parental encouragement and administrative commitment further reinforced program acceptance, underscoring the importance of inclusive, multi-level stakeholder participation.

For sustained implementation and long-term impact, eSHEPP must be institutionalized within the education system through supportive policy frameworks, comprehensive teacher training, and investment in ICT infrastructure. Addressing digital inequities, especially in underserved and rural areas, will be critical to ensuring equitable access and inclusiveness. With ongoing refinement, strong policy support, and systematic integration, eSHEPP could significantly enhance adolescent health literacy and inform national and regional strategies for NCD prevention. These findings also offer transferable lessons for other LMICs aiming to embed digital health education within school systems.

## Supplementary Information

Supplementary Material 1.

Supplementary Material 2.

Supplementary Material 3.

Supplementary Material 4.

Supplementary Material 5.

## Data Availability

The deidentified interview transcripts analyzed during the current study are not publicly available due to confidentiality concerns but may be made available from the corresponding author upon reasonable request and subject to approval from the ethics committee.

## Abbreviations

AKU: Aga Khan university
COREQ: Consolidated criteria for reporting qualitative research
DEO: District education officer
FGD: Focus group discussion
ICT: Information and communication technology
KII: Key informant interview
LMICs: Low- and middle-income countries
NCDs: Noncommunicable diseases
eSHEPP: School eHealth education program Pakistan
SMC: School management committee
SLO: Student learning outcome
TTF: Task–technology fit
TAM: Technology acceptance model
WHO: World health organization
