# Supplementary material for: School eHealth education program Pakistan (eSHEPP): an exploratory qualitative study of stakeholder perspectives on design, barriers, and facilitators
Source: J Health Popul Nutr. 2025 Nov 26;44:432. doi: 10.1186/s41043-025-01170-0 (PMC12752358; doi:10.1186/s41043-025-01170-0)
Supplement: Supplementary file 1 — Supplementary Material 1. [file 41043_2025_1170_MOESM1_ESM.docx]

**Supplementary File 1 - Codebook Audit and Reliability Documentation**

**Table S1. Iterative Development of the Codebook**

| **Version** | **Key Changes / Developments** | **New Codes Added** | **Codes Modified** | **Codes Removed** | **Rationale** |
| --- | --- | --- | --- | --- | --- |
| **First** | Initial code extraction from qualitative data | - Familiarity with Digital eHealth Apps - Technology Acceptance - Utilization of Digital eHealth Apps - Multimedia Delivery Acceptance - Cultural or Social Barriers - Digital Literacy Skills | N/A | N/A | Initial broad coding based on raw qualitative data |
| **Second** | Reorganization and refinement of thematic areas | - Features of the Application for Enhanced Learning Experience - Interactive Features in the Application - Enhancing User Experience & Effectiveness | - Combined “Effectiveness & Potential Benefits” - Expanded “Engagement” concepts | Removed “Program Implementation Success Factors” (merged with Facilitators) | First thematic grouping attempt |
| **Third** | Introduction of structural hierarchy with parent–child codes | - Facilitators (Enablers of Success) [Parent Code] - Prior Experience with eHealth Tools - Teacher Training and Capacity Building | - “Anticipated Challenges” expanded with child codes - “Design of eHealth Application” restructured with sub-codes | Removed “Real-Life Examples & Personal Stories” (merged with Video Content) | Early theoretical framework development |
| **Fourth** | Formalization of parent–child relationships | - Motivation and Engagement Strategies [Parent Code] - Student Preferences & Expectations [Parent Code] | - “Stakeholder Involvement” refined with specific child codes - “Program Acceptance” expanded | Removed “Enhancing Engagement & Effectiveness” (redundant) | Streamlining code relationships and removing redundancy |
| **Fifth** | Final thematic consolidation | - Implementation Challenges and Enablers [Parent Code] - Program Design and Content [Parent Code] - Stakeholder Engagement and Involvement [Parent Code] | - “Facilitators” renamed to “Facilitating Factors” - “Perceived Benefits” expanded with application / video specifics | Removed “Prior Experience” (merged with Digital Readiness) | Consolidation to support theoretical alignment |
| **Final** | Comprehensive thematic structure with finalized hierarchy | - Program Acceptance and Digital Readiness [Parent Code] - Perceived Benefits and Motivation [Parent Code] | - All child codes reviewed and standardized - “Facilitator’s Role” moved to standalone child code | Removed “Teacher Training” (merged with Stakeholder Engagement) | Final consolidation after full team review and alignment with conceptual frameworks |

**Table S2. Final Codebook Refinement Decisions**

| **Original Code (Early Versions)** | **Final Codebook Placement** | **Modification Rationale** | **Team Decision Process** |
| --- | --- | --- | --- |
| Familiarity with Digital eHealth Apps | 4.3 Previous Experience with Digital Tools | Reframed for broader conceptual fit with digital readiness | Unanimous agreement after team review |
| Technology Acceptance | 4.1.1 Acceptance of Technology in Health Education | Adopted more precise terminology to reflect health education focus | Three iterative rounds of team discussion |
| Utilization of Digital eHealth Apps | 2.1 Design of the eHealth Application | Merged into design-related codes to reduce overlap and improve clarity | Consensus decision within the team |
| Multimedia Delivery Acceptance | 4.1.2 Attitudes Toward Multimedia-Based Learning | Refined into a more descriptive and actionable label | Suggested and agreed upon during team deliberation |
| Cultural or Social Barriers | 1.1 Anticipated Challenges (implied) | Incorporated into broader code on anticipated challenges to avoid redundancy | Team agreed explicit code was unnecessary |
| Digital Literacy Skills | 4.2 Student Comfort and Literacy with Digital Tools | Reframed positively to emphasize student capabilities rather than deficits | Decision reached after three rounds of team discussion |
| Program Implementation Success Factors | 1.2 Facilitating Factors | Adjusted to align with terminology commonly used for enablers/supportive factors | Unanimous agreement within the team |
| Teacher & School Staff Involvement | 3.3 Teacher and Administrator Involvement / 3.4 Teacher Training for Sustainability | Split into two codes for more detailed and nuanced analysis | Recommended by team members with methodological expertise and accepted collectively |
| Parental/Guardian Involvement | 3.1 Parental or Guardian Involvement | Standardized terminology for consistency across the codebook | Agreed by team in alignment with internal style guide |
| Features for Enhanced Learning Experience | 2.1.1 Engaging Application Features | Streamlined for clarity and ease of coding | Finalized following a team card-sorting exercise |
| User-Friendly Design | 2.1.2 User Interface and Usability | Broadened to capture overall usability aspects beyond design simplicity | Adopted after input from team members with user-experience expertise |
| Relevant Topics | 2.2.3 Relevance of Health Topics | Clarified scope to focus specifically on topic appropriateness for students | Decision reached collectively by the content-focused subgroup |
| Effectiveness & Potential Benefits | 5.1 Perceived Benefits | Realigned to fit more closely with the Technology Acceptance Model (Perceived Usefulness) | Adopted after methodological team discussions |
| Engagement of Participants | 1.2.3 Student Interest and Participation | Refined into a more specific and measurable label | Adjusted following inter-rater reliability testing and team feedback |
| Facilitator’s Role and Challenges | 1.3 Facilitator’s Role | Simplified label for consistency with other codes | Streamlined during the final team review process |

**Audit Notes**

- The codebook was refined through five iterative cycles over a three-month period, supported by weekly team review meetings.
- Three team members provided targeted peer feedback between Versions 3 and 4 to enhance clarity and consistency.
- The final codebook structure was mapped to the integrated framework of the Technology Acceptance Model (TAM) and the Task–Technology Fit (TTF) Model.
- Intercoder reliability testing was conducted prior to finalization to ensure consistency in code application.

**Table S3. Intercoder Reliability Testing**

| **Measure of Agreement** | **Value** | **Std. Error** | **Z** | **Sig.** | **Benchmark (Landis & Koch, 1977)** |
| --- | --- | --- | --- | --- | --- |
| Cohen’s Kappa | 0.7097 | 0.1976 | 3.591 | <0.001 | Substantial Agreement (0.61–0.80) |

**Interpretation:** The intercoder reliability testing demonstrated a high level of consistency between coders. As shown in Table 3, the agreement between Reviewer A and Reviewer B yielded a Cohen’s Kappa of 0.710 (p < .001), indicating substantial agreement. According to the benchmark proposed by Landis and Koch (1977), κ values between 0.61 and 0.80 reflect substantial agreement, suggesting that the coding scheme was applied reliably across reviewers.
