# Supplementary material for: School eHealth education program Pakistan (eSHEPP): an exploratory qualitative study of stakeholder perspectives on design, barriers, and facilitators
Source: J Health Popul Nutr. 2025 Nov 26;44:432. doi: 10.1186/s41043-025-01170-0 (PMC12752358; doi:10.1186/s41043-025-01170-0)
Supplement: Supplementary file 3 — Supplementary Material 3. [file 41043_2025_1170_MOESM3_ESM.docx]

**Supplementary File 2. School eHealth Education Program Pakistan - Codebook**

| **Name** | **Description (short)** | **Reference (Parent Node → Framework Construct)** |
| --- | --- | --- |
| 1. Implementation Challenges and Enablers | Captures both barriers and supportive factors affecting implementation. | TTF: Organizational / contextual support; TTF: Task–Technology fit (alignment of task & technology) |
| - 1. Barriers | Main obstacles that hinder implementation/adoption. |  |
| - - 1. Anticipated Challenges | Resistance, institutional issues, contextual barriers. |  |
| - - 1. Infrastructure – Electricity, Internet, Multimedia | Availability/reliability of electricity, internet, multimedia equipment. |  |
| - 1. Facilitating Factors | Enablers: policies, resources, trained staff, stakeholder buy-in. |  |
| - - 1. Access to Devices and Connectivity | Access to smartphones, internet, electricity. |  |
| - - 1. Parental Awareness and Engagement | Parental knowledge/participation supporting the program. | Open-coded |
| - - 1. Student Interest and Participation | Student motivation, engagement, and active involvement. |  |
| - - 1. Supportive Attitudes of Teachers and Administrators | Willingness of teachers/admins to integrate the program. |  |
| - - 1. Teacher Training for Sustainability | Building teacher capacity for long-term delivery. | Open-coded |
| - 1. Facilitator’s Role | Challenges and contributions of program facilitators. |  |
| 1. Perceived Benefits | Participants’ reported positive impacts of the program. | TAM: Perceived usefulness; TAM: Perceived ease of use |
| - 1. Perceived Benefits – eHealth Application | Advantages of the app (learning, accessibility). |  |
| - 1. Perceived Benefits – Health Promoting Videos | Benefits of videos for NCD awareness/learning. |  |
| - 1. Perceived Benefits – Overall Program | Overall program impact on student health awareness. |  |
| 1. Program Acceptance and Digital Readiness | Stakeholder readiness and openness to digital health education. | TAM: Attitude toward use; TTF: Task–Technology fit (alignment of task & technology) |
| - 1. Previous Experience with Digital Tools | Prior exposure and comfort using digital tools. |  |
| - 1. Program Acceptance | General attitudes and willingness to adopt the program. |  |
| - 1. Acceptance of Technology in Health Education | Willingness to use tech-based health education solutions. |  |
| - 1. Attitudes Toward Multimedia-Based Learning | Receptivity to using video/animation in class. |  |
| - 1. Student Comfort and Literacy with Digital Tools | Students’ digital skills and navigational ability. |  |
| 1. Program Design and Content | Technical, visual and educational quality of tools/content. | TTF: Technology characteristics / Technical fit; TAM: Perceived ease of use |
| - 1. Design of the eHealth Application | Functional/technical design ensuring usability/relevance. |  |
| - - 1. Engaging Application Features | Interactive components that enhance learning (quizzes, games). |  |
| - - 1. User Interface and Usability | Need for intuitive, accessible UI for varied digital skills. |  |
| - - 1. App Usability Concerns from Stakeholders | Stakeholder worries about ease of use, access, content. |  |
| - 1. Health Promoting Videos | Use of videos as a delivery medium for health content. |  |
| - - 1. Language Preferences | Preferred languages for content delivery and inclusion. |  |
| - - 1. Optimal Video Length | Preferred duration balancing attention and learning. |  |
| - - 1. Relevance of Health Topics | Fit of topics (NCDs, nutrition, activity) to student needs. |  |
| - - 1. Video Content and Structure | Storytelling, visuals, sequence and clarity of videos. |  |
| - 1. Motivation and Engagement Strategies | Ways to sustain student motivation and participation. | Open-coded |
| 1. Stakeholder Engagement and Involvement (parent) | Roles of parents, teachers, admins, policymakers. | TTF: Organizational / contextual support |
| - 1. Parental or Guardian Involvement | Parent support to reinforce participation and uptake. |  |
| - 1. Student Preferences & Expectations | Desired features, formats, interactivity from students. |  |
| - 1. Teacher and Administrator Involvement | Teachers/admins’ contribution to integration and sustainability. |  |

Abbreviations: TAM = Technology Acceptance Model TTF = Task–Technology Fit
