# Supplementary material for: School eHealth education program Pakistan (eSHEPP): an exploratory qualitative study of stakeholder perspectives on design, barriers, and facilitators
Source: J Health Popul Nutr. 2025 Nov 26;44:432. doi: 10.1186/s41043-025-01170-0 (PMC12752358; doi:10.1186/s41043-025-01170-0)
Supplement: Supplementary file 4 — Supplementary Material 4. [file 41043_2025_1170_MOESM4_ESM.docx]

**Supplementary File 5 - Focus group and key informant interview guides**

This file contains the full guides used for focus group discussions with students and teachers, and key informant interviews with parents and school administrators. These guides include main questions, probes, and instructions for moderators/interviewers.

**Focus Group Discussion guide:**

School eHealth Education Program Pakistan to Improve Awareness in Adolescents from Urban Pakistan

**Guidelines for Focus Group Discussion (FGD) with students and teachers.**

**Consent**: Written informed consent will be signed by each participant before commencing the FGD.

**Duration**: Nearly 45 minutes will be allocated, or it can be extended until the point of saturation

**Mode of recording**: Tape recorder will be used for recording. In addition, written notes will also be taken.

**Place for FGD**: A secure and comfortable space within the school premises, such as a school library or study room where participants feel comfortable, and their privacy will be ensured.

**Transcription**: Following the completion of each discussion, tape verbatim will be transcribed, noting pauses, changes in tone, laughter, and moderator’s questions, comments, and affirmative “noises.” In addition, length of FGD and amount of time required to transcribe will also be noted at the end of transcript, so that other FGDs can be modified or implemented accordingly.

FGD will be conducted in a team of two. One person will moderate the session, and the other will record the responses, both in writing and by audio recorder.

**General instructions**

- **Welcome the participants**
- **Overview of the topic**

The focus of the School eHealth Education Program Pakistan is to promote healthy behaviors among students, specifically related to diet, physical activity, and other factors associated with NCD prevention. The program will be delivered through a digital application accessible via internet browsers, which can be utilized within the school campus if necessary. As part of this study, health education videos will be incorporated into classroom sessions using multimedia. Each session will consist of 25 to 30 students, with a trained facilitator guiding the discussions during the session.

- **Purpose of the FGD**

The purpose of the FGD is to explore participants’ views regarding the barriers and facilitators associated with implementing the School eHealth Education Program Pakistan in schools. Furthermore, the participants' viewpoints on the content and design of the program will also be explored.

- **Ground rules of FGD**
- Talk one at a time in a voice as loud as mine.
- Respect each other’s opinions.
- Avoid side conversations with those sitting next to you.
- The group needs to hear from everyone during the discussion, but you are not obliged to answer every question.
- Only respond to questions that you can relate to and feel comfortable in answering.
- Feel free to respond directly to someone who has made a point. You do not have to address your comments to the facilitator in-order for them to be recorded.
- Say what is true for you.
- Ask questions/clarification as they come up.

FGD session No: ________________

**Session attendance information sheet** (To be filled by participants)

| **S. No** | **Name of student / teacher** | **Age (years)** | **Class / Teaching experience in yrs.** | **Contact details** | **Written consent** |
| --- | --- | --- | --- | --- | --- |
| 1. |  |  |  |  |  |
| 2. |  |  |  |  |  |
| 3. |  |  |  |  |  |
| 4. |  |  |  |  |  |
| 5. |  |  |  |  |  |
| 6. |  |  |  |  |  |

**To be filled by moderator:**

Date of FGD: __/__/____ Duration of FGD: ________

Place of FGD: Begin - __: __

Name of moderator: End - __: __

Name of note

| **S. No.** | **PROBE** | **COMMENTS** |
| --- | --- | --- |
|  | Thoughts on digital applications to improve health and wellbeing of adolescents.  Probe: Familiarity with digital eHealth apps, Technology acceptance , Utilization of digital eHealth apps |  |
|  | Opinions on incorporating health education videos into classroom sessions using multimedia.  Probe: Acceptance of Multimedia Delivery, Perceived Benefits of Health Education Videos, Students’ Preferences and Expectations |  |
|  | Relevance and applicability of the program  Probe: Views on the effectiveness and potential benefits of the program. Interest and engagement of the participants in the program. |  |
| **Barriers to Implementation of the eSHEPP** | | |
|  | Anticipated challenges in implementing the eHealth NCD prevention program in schools.  Probe: Potential difficulties and obstacles that may arise during the implementation of the eHealth NCD prevention program in educational institutions. |  |
|  | Cultural or social barriers.  Probe: Factors pertaining to culture or society that could hinder the successful implementation of the program. |  |
|  | Students’ digital literacy skills  Probe: Students level of digital literacy skills. Potential difficulties or challenges in using technology |  |
|  | Students’ motivation and sustained engagement:  Probe: Challenges in terms of students' motivation and engagement in the program and strategies to overcome these challenges. |  |
| **Facilitators to Implementation of the eSHEPP** | | |
|  | Program Implementation Success Factors  Probe: Factors contributing to the successful implementation of the program. Key elements that facilitate program success and effectiveness. |  |
|  | Teacher and School Staff Involvement  Probe: The role of teachers and school staff in delivering the program effectively. How their participation enhances program outcomes and student experiences? |  |
|  | Involvement of Parents or Guardians  Probe: The potential benefits of involving parents or guardians in the program. How can their participation support students and contribute to program success? |  |
|  | Facilitator's Role and Challenges  Probe: The role of the facilitator in delivering the program. Challenges they may encounter during classroom sessions and strategies to address these challenges. |  |
| **Content of the Health promoting videos:** | | |
|  | Topics to include in health education videos  Probe: Relevant topics related to diet, physical activity, and NCD prevention that should be included in the videos. Key areas of focus for comprehensive health education are on NCDs prevention. |  |
|  | Importance of Real-Life Examples and Personal Stories  Probe: The significance of incorporating real-life examples or personal stories in the videos. How can such content enhance the relevance and impact of the health education program? |  |
|  | Enhancing Engagement and Effectiveness of Health Education Videos  Probe: Strategies to make health education videos more engaging and effective for students. Techniques to capture attention, maintain interest, and promote active learning. |  |
|  | Length and Format of videos  Probe: Suggestions for the ideal length and presentation of videos to ensure they are engaging and informative. Considerations for optimal delivery and retention of information by students. |  |
| **Design of the eHealth app:** | | |
|  | Digital Application Features for Enhanced Learning Experience  Probe: Specific features or interactive components that would enhance students' learning experience and motivation within the digital application. Key elements that promote active participation and knowledge retention. |  |
|  | Designing a User-Friendly Application:  Probe: Strategies to ensure ease of use for all students, including those with limited digital literacy skills. Design considerations for a user-friendly interface. |  |
|  | Interactive Features for Enhanced User Engagement  Probe: Types of interactive features or functionalities that should be incorporated into the application to enhance user engagement. Methods to keep students actively involved and interested in the content. |  |
|  | Enhancing User Experience and Program Effectiveness:  Probe: Other features or design elements that might enhance the overall user experience and effectiveness of the health education program. |  |

We have reached the end of our discussion. Thank you for participating.

**Key Informant Interview guide:**

School eHealth Education Program Pakistan to Improve Awareness in Adolescents from Urban Pakistan

**Guidelines for Key Informant Interview (KII) with parents and school administrators.**

**Consent**: The participant will sign written consent form before commencing each key informant interview.

**Duration**: Nearly 30 minutes will be allocated, or it can be extended until the point of saturation

**Mode of recording**: Tape recorder will be used for recording. In addition, written notes will also be taken.

**Place for Interview**: Any suitable place in the educational institute facility/ department of education will be chosen as per the participant’s comfort. Prime importance will be given to the place where the participant is comfortable, and his/her privacy is ensured.

**Transcription**: Following the interview, tape verbatim will be transcribed, noting pauses, changes in tone, laughter, comments, and affirmative “noises.” In addition, the length of the interview and amount of time required to transcribe will also be noted at the end of the transcript, so that other key informant interviews can be modified or implemented accordingly.

The interview will be conducted in a team of two. One person will ask the questions, and the other will record the responses, both in writing and by audio recorded.

**General instructions**

- **Welcome the participant**
- **Overview of the topic**

The focus of the School eHealth Education Program Pakistan is to promote healthy behaviors among students, specifically related to diet, physical activity, and other factors associated with NCD prevention. The program will be delivered through a digital application accessible via internet browsers, which can be utilized within the school campus if necessary. As part of this study, health education videos will be incorporated into classroom sessions using multimedia. Each session will consist of 25 to 30 students, with a trained facilitator guiding the discussions during the session.

- **Purpose of the KII**

The purpose of the KII is to explore participant’s views regarding the barriers and facilitators associated with implementing the School eHealth Education Program Pakistan in schools. Furthermore, the participant’s viewpoints on the content and design of the program will also be explored.

- **Ground rules of KII**
- Talk in a voice as loud as mine.
- Only respond to questions that you can relate to and feel comfortable in answering.
- Say what is true for you.
- Ask questions/clarification as they come up.

KII session No: ________________

**PARTICIPANT’S INFORMATION: to be filled by participant.**

| Name of participant |  |
| --- | --- |
| Designation |  |
| Place of work or institution |  |
| Work experience |  |
| Qualification |  |
| Contact details |  |
| Written consent | Yes / No |
| Signature/Thumb Impression |  |

**To be filled by interviewer**

| Name of Interviewer |  |
| --- | --- |
| Name of note-taker |  |
| Duration of interview | Begin End |
| Date of Interview | DD / MM/ YY |

| **S. No.** | **PROBE** | **COMMENTS** |
| --- | --- | --- |
|  | Thoughts on digital applications to improve health and wellbeing of adolescents.  Probe: Familiarity with digital eHealth apps, Technology acceptance , Utilization of digital eHealth apps |  |
|  | Opinions on incorporating health education videos into classroom sessions using multimedia.  Probe: Acceptance of Multimedia Delivery, Perceived Benefits of Health Education Videos, Students’ Preferences and Expectations |  |
|  | Relevance and applicability of the program  Probe: Views on the effectiveness and potential benefits of the program. Interest and engagement of the participants in the program. |  |
| **Barriers to Implementation of the eSHEPP** | | |
|  | Anticipated challenges in implementing the eHealth NCD prevention program in schools.  Probe: Potential difficulties and obstacles that may arise during the implementation of the eHealth NCD prevention program in educational institutions. |  |
|  | Cultural or social barriers.  Probe: Factors pertaining to culture or society that could hinder the successful implementation of the program. |  |
|  | Students’ digital literacy skills  Probe: Students level of digital literacy skills. Potential difficulties or challenges in using technology |  |
|  | Students’ motivation and sustained engagement:  Probe: Challenges in terms of students' motivation and engagement in the program and strategies to overcome these challenges. |  |
| **Facilitators to Implementation of the eSHEPP** | | |
|  | Program Implementation Success Factors  Probe: Factors contributing to the successful implementation of the program. Key elements that facilitate program success and effectiveness. |  |
|  | Teacher and School Staff Involvement  Probe: The role of teachers and school staff in delivering the program effectively. How their participation enhances program outcomes and student experiences? |  |
|  | Involvement of Parents or Guardians  Probe: The potential benefits of involving parents or guardians in the program. How can their participation support students and contribute to program success? |  |
|  | Facilitator's Role and Challenges  Probe: The role of the facilitator in delivering the program. Challenges they may encounter during classroom sessions and strategies to address these challenges. |  |
| **Content of the Health promoting videos:** | | |
|  | Topics to include in health education videos  Probe: Relevant topics related to diet, physical activity, and NCD prevention that should be included in the videos. Key areas of focus for comprehensive health education are on NCDs prevention. |  |
|  | Importance of Real-Life Examples and Personal Stories  Probe: The significance of incorporating real-life examples or personal stories in the videos. How can such content enhance the relevance and impact of the health education program? |  |
|  | Enhancing Engagement and Effectiveness of Health Education Videos  Probe: Strategies to make health education videos more engaging and effective for students. Techniques to capture attention, maintain interest, and promote active learning. |  |
|  | Length and Format of videos  Probe: Suggestions for the ideal length and presentation of videos to ensure they are engaging and informative. Considerations for optimal delivery and retention of information by students. |  |
| **Design of the eHealth app:** | | |
|  | Digital Application Features for Enhanced Learning Experience  Probe: Specific features or interactive components that would enhance students' learning experience and motivation within the digital application. Key elements that promote active participation and knowledge retention. |  |
|  | Designing a User-Friendly Application:  Probe: Strategies to ensure ease of use for all students, including those with limited digital literacy skills. Design considerations for a user-friendly interface. |  |
|  | Interactive Features for Enhanced User Engagement  Probe: Types of interactive features or functionalities that should be incorporated into the application to enhance user engagement. Methods to keep students actively involved and interested in the content. |  |
|  | Enhancing User Experience and Program Effectiveness:  Probe: Other features or design elements that might enhance the overall user experience and effectiveness of the health education program. |  |

We have reached the end of our interview. Thank you for participating.
