# Supplementary material for: School eHealth education program Pakistan (eSHEPP): an exploratory qualitative study of stakeholder perspectives on design, barriers, and facilitators
Source: J Health Popul Nutr. 2025 Nov 26;44:432. doi: 10.1186/s41043-025-01170-0 (PMC12752358; doi:10.1186/s41043-025-01170-0)
Supplement: Supplementary file 5 — Supplementary Material 5. [file 41043_2025_1170_MOESM5_ESM.docx]

**Supplementary File 3 - Table S4 - S7 & Figure S1 - S2**

**Participant Demographics (KIIs and FGDs)**

**Table S4. Parent Participant Demographics - KIIs**

| Participant ID | Qualification | Employment | Age (years) | Gender |
| --- | --- | --- | --- | --- |
| Parent 1 | Postgraduate | Driver | 51 | Male |
| Parent 2 | Matric | Driver | 52 | Male |
| Parent 3 | Graduate | Nurse | 42 | Female |
| Parent 4 | Postgraduate | Health Technologist | 51 | Female |

**Table S5. School Administrator Participant Demographics - KIIs**

| Participant ID | Age (years) | Designation | Gender |
| --- | --- | --- | --- |
| Administrator 1 | 59 | Senior Headmistress | Female |
| Administrator 2 | 55 | Deputy Director | Male |
| Administrator 3 | 53 | Senior Headmistress | Female |
| Administrator 4 | 48 | District Education Officer | Male |
| Administrator 5 | 52 | Principal | Male |
| Administrator 6 | 59 | Principal | Male |
| Administrator 7 | 47 | Deputy Director | Female |

**Table S6. Student Participant Demographics - FGDs**

| Group | Gender | No. of Participants | Mean Age (years) |
| --- | --- | --- | --- |
| FGD-S1 | Male | 10 | 14.9 |
| FGD-S2 | Female | 10 | 14.5 |

**Table S7. Teacher Participant Demographics – FGDs**

| Group | Gender | No. of Participants | Mean Age (years) | Mean Teaching Experience (years) |
| --- | --- | --- | --- | --- |
| FGD-T1 | Male | 8 | 43.5 | 21.75 |
| FGD-T2 | Female | 8 | 41.5 | 14.81 |


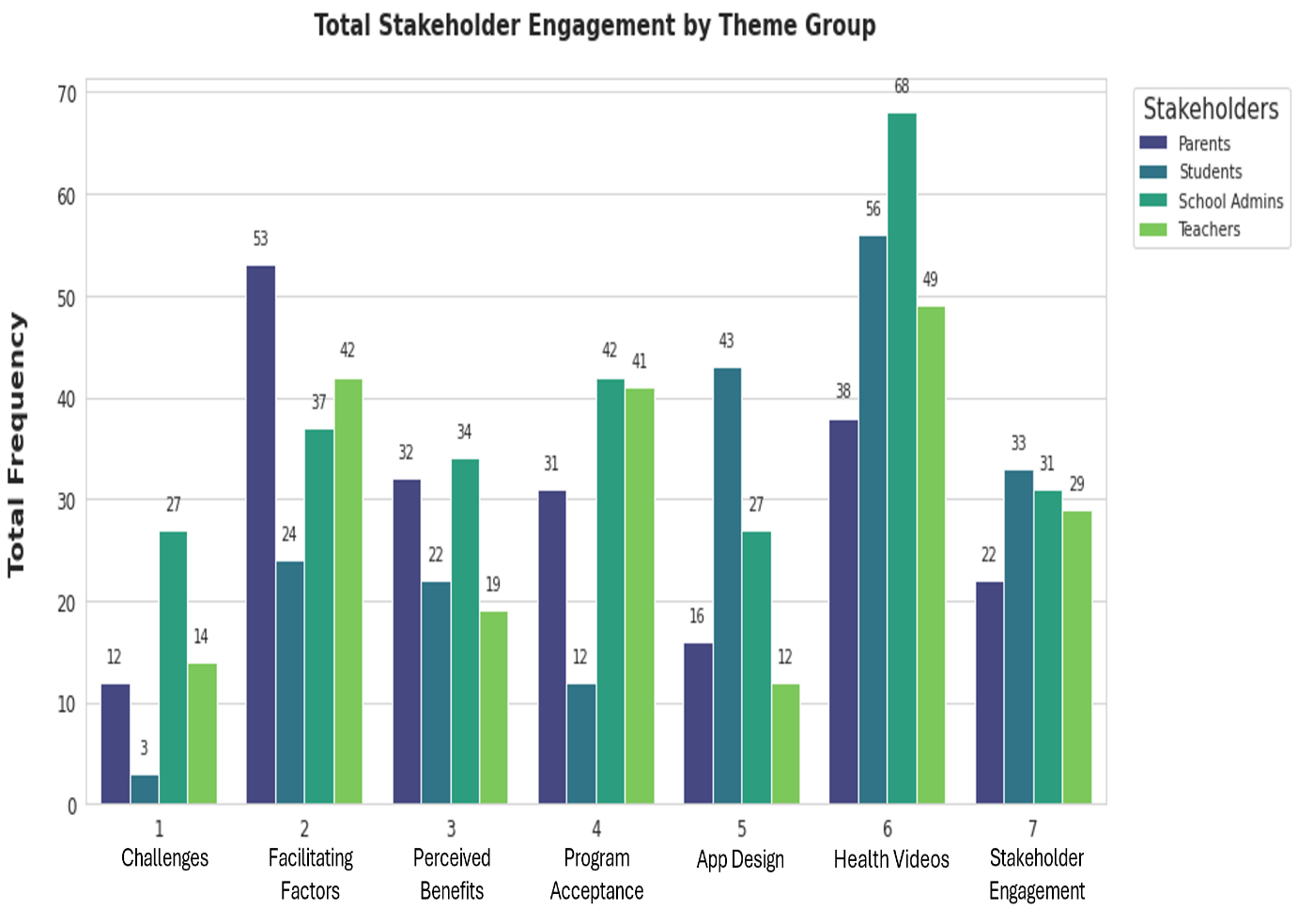


*Figure S1: Distribution of Stakeholder Engagement Across Major Themes*


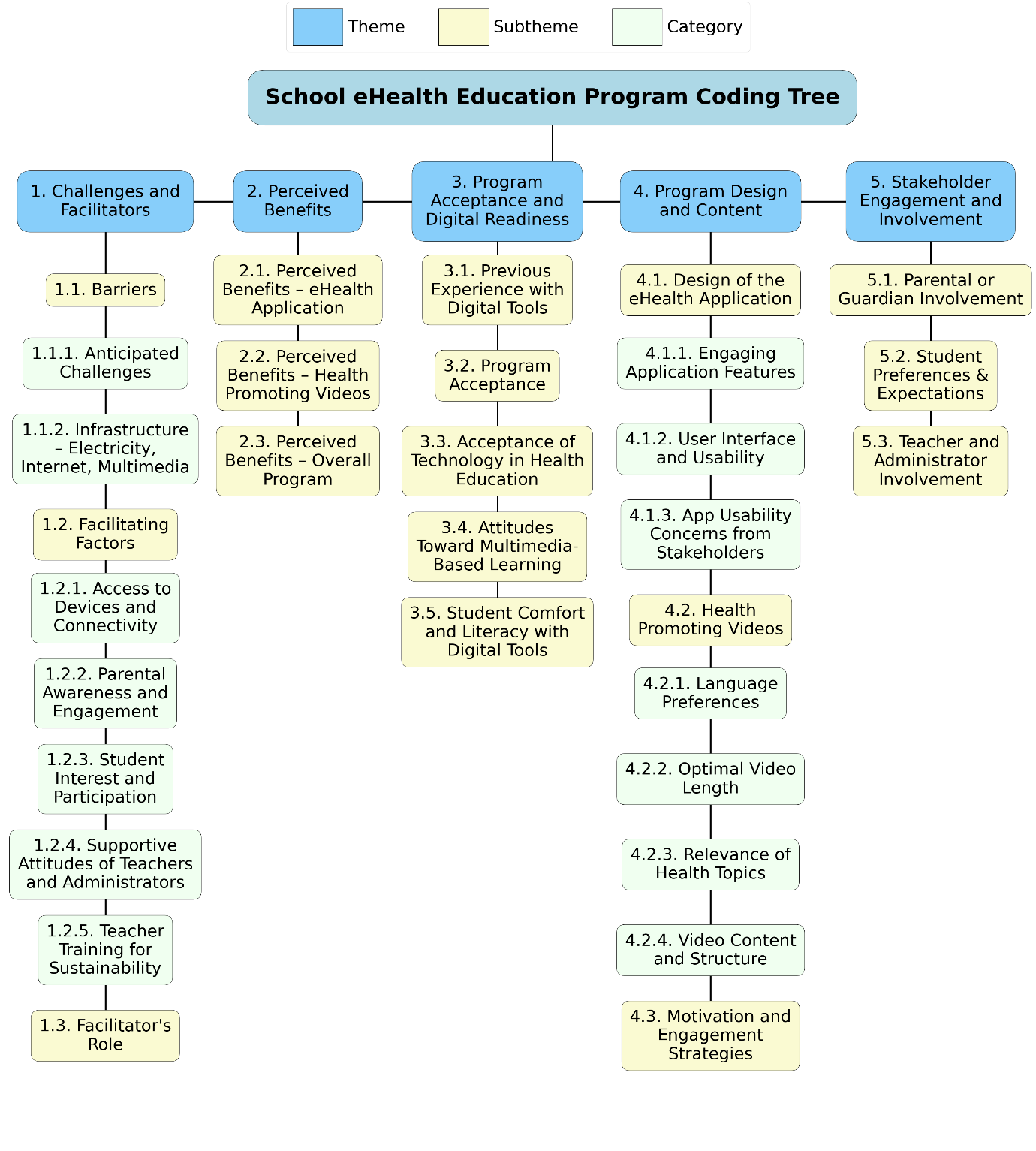


*Figure S2: eSHEPP Coding tree of themes, subthemes, and categories*
